# The Chinese Communist Party and regulatory transparency in China's food industry

**DOI:** 10.1093/pnasnexus/pgad028

**Published:** 2023-02-03

**Authors:** Qihua Gao, Yasheng Huang, Yuze Sui, Yanchong Zheng

**Affiliations:** Sloan School of Management, Massachusetts Institute of Technology, 100 Main Street, E62-462, Cambridge, MA 02142, USA; Sloan School of Management, Massachusetts Institute of Technology, 100 Main Street, E62-462, Cambridge, MA 02142, USA; Department of Sociology, School of Humanities and Sciences, Stanford University, 450 Jane Stanford Way, Building 120, Stanford, CA, USA; Sloan School of Management, Massachusetts Institute of Technology, 100 Main Street, E62-462, Cambridge, MA 02142, USA

**Keywords:** regulatory transparency, food safety, data-driven method, China, party system, administrative system

## Abstract

While it is widely accepted that the Chinese Communist Party (CCP) occupies a dominant position in the Chinese political system, few studies have demonstrated CCP's dominant position based on rigorous statistical analysis. Our paper presents the first such analysis using an innovative measure of regulatory transparency in the food industry across nearly 300 prefectures in China over 10 years. We show that actions by the CCP, while broadly scoped and not targeting the food industry, significantly improved regulatory transparency in the industry. In sharp contrast, food-industry-specific interventions by the State Council, which exercises direct regulatory supervision of the industry, had no impact on regulatory transparency. These results hold in various specifications and robustness checks. Our research contributes to research in China's political system by empirically and explicitly demonstrating the dominating power of the CCP.

**Significance Statement**

This paper presents the first rigorous, statistical demonstration of the Chinese Communist Party (CCP)'s dominant position in the Chinese political system. Using an innovative measure of regulatory transparency in the food industry across nearly 300 prefectures in China over 10 years, we show that actions by the CCP, while broadly scoped and not targeting the food industry, significantly improved regulatory transparency in the industry. In sharp contrast, food-industry-specific interventions by the State Council, which exercises direct regulatory supervision of the industry, had no impact on regulatory transparency. Our findings suggest that the CCP has a unique capacity to shape regulatory developments in food safety, and that potentially beneficial changes, such as improving transparency, require actions by the CCP.

## Introduction

It is generally asserted that China is ruled by the Chinese Communist Party (CCP). China's President, Xi Jinping, derives his ruling power from being the CCP leader—the general secretary of the CCP, not from the largely ceremonial post of presidency. In government organizations, enterprises, and even universities, the CCP exercises its control primarily through personnel appointments. The secretaries of the CCP outrank ministers, general managers, or chancellors (1).

Despite such widely accepted claims, few studies have empirically tested the existence and consequence of CCP's dominant position in the Chinese political system with a data-driven approach. This paper fills this gap. Specifically, we use a statistical approach to demonstrate the power and impact of the CCP in an area of central importance to the Chinese society—food safety regulation. Does the CCP play a unique and direct role in regulating the safety of China's food? Can we use data-driven methods to prove or disprove the above claim? To answer these questions, we contrast the role of CCP in food safety regulation with that of the State Council, the apex of government organization which directly supervises the food regulatory agency—China Food and Drug Administration (CFDA).

Our research shows that the power of the CCP in China's food safety regulation substantially exceeds that of the State Council. The CCP's actions, while general in scope and not targeting the food industry, have a significant impact on the level of regulatory transparency in the food industry. In sharp contrast, actions of the State Council that specifically target the food industry do not have a statistically significant impact on regulatory transparency in the industry.

We use the auditing inspections—conducted by the Central Commission for Discipline Inspection (CCDI) under the CCP—as our measure of the intervention actions of the CCP. These auditing inspections are designed to examine the general conduct of the officials. They are not issue specific and they are not targeted against food safety. We contrast this general intervention by the CCP with an action by the State Council specifically targeting food safety—establishment of provincial Food Safety Commissions (FSCs). We found that auditing inspections by the CCDI significantly increased the transparency scores of prefectural CFDAs whereas the establishments of FSCs did not.

Our findings confirm the current qualitative literature on the CCP: CCP is powerful and its power can exceed that of the State Council, at least in the area of regulatory transparency in food safety. Importantly, our results advance the current literature in two ways. First, while the CCP is generally known for its opacity and secrecy, in the particular area of food safety, the impact of the CCP is surprisingly an increase of transparency. Why this is the case is beyond the scope of our paper and calls for further research. Second, by design, our statistical analysis controls for the personnel effect of the CCP—the leaders of the CFDAs and FSCs are all appointed by the CCP.^1^ Therefore, any differential impacts of the CCP versus State Council actions on CFDA transparency that we observe can be plausibly attributed to the direct operational effect of the CCP rather than to its personnel effect alone. This finding goes beyond much of the current literature that emphasizes the personnel effect of the CCP.

We develop an innovative measure of regulatory transparency in food safety across nearly 300 prefectures in China between 2008 and 2017. (In 2018, the CFDA was merged with other government agencies into a new agency called the State Administration for Market Regulation, SAMR. The data are not comparable before and after 2018. Hence, we restrict our analysis to the period before 2018.) This large-scale, systematic, first-of-its-kind transparency scoring is done using advanced text analytics that combines web scraping, image recognition, and string-matching algorithms. Our statistical analysis leverages a set of exogenous shocks to the food industry to capture the CCP's regulatory intervention and to contrast its impact on food regulatory transparency versus the impact of the State Council's intervention.

Our analysis breaks new ground in a number of areas. First, we show the supremacy of the CCP in ruling China, a claim often asserted but rarely demonstrated empirically by social scientists. Furthermore, our results suggest that this supremacy of the CCP potentially goes beyond the CCP's personnel control (2).^2^ To the best of our knowledge, our paper is the first to examine the impact of the CCP on food safety in China and to explicitly benchmark the CCP's action against the State Council's action. Our analysis shows the stronger impact of CCP's general-scoped actions on food safety regulations, when compared with food-safety-specific actions by the State Council.

Second, we advance the state of research on food safety regulation in China by performing statistical analysis on novel and granular regulatory data that did not exist before. China's problematic food safety record has attracted a large body of research on the topic. However, much of this research is qualitative, relying on perceptional surveys, textual interpretations of China's regulatory structure and its progression, and a few specific case studies as the empirical basis for analysis (e.g. (3–13)). While this research is rich and informative, the findings are often at a very high level, sometimes treating the entire country as a single data point. Quantitative policy research related to Chinese food safety focuses more on the consumer side rather than on the regulatory side of the issue (14). This is understandable: Regulatory data are extremely difficult to access or collect. The regulatory data we have constructed complement existing research by exploring and examining regulatory heterogeneities among nearly 300 (of 333 total) prefectures in China over a 10-year span.

Third, our regulatory transparency measures—created according to features of the prefectural CFDA websites—are based on observations of objective outcomes and are granular and systematic, both spatially and temporally, when compared with prior research based on analysis of regulatory intent. The measures we have developed can be regularly updated via an automated algorithm, thus enabling continuous assessment over time, overcoming the access and intermittency problems of survey research on China.

Fourth, the topic of regulatory transparency is missing in current research on Chinese food safety. The rising middle class in China and the increasing sophistication of consumer preference and choice mean that regulatory effectiveness is no longer only a function of government power but also of the awareness and knowledge of Chinese consumers. Our transparency measure is thus bidirectional, capturing both the government's actions of conveying food safety information to the public and the ability of the public to report food safety incidents to the government. Although this is beyond the scope of our paper, we believe that transparency has a meaningful effect on regulatory outcome, as research in non-China contexts indicates (e.g. (15–17)).

The remainder of the paper proceeds as follows. First, we introduce our measurement of regulatory transparency in Chinese food safety and our methodology to construct the prefectural CFDA transparency scores. Second, we explain the two main independent variables, interventions by the CCP and the State Council. We then present our statistical results and conclude with broad analytical and policy implications.

### Regulatory transparency of prefectural CFDA branches

The CFDA has a local branch in every prefecture in China. Our analysis focuses on the regulatory transparency of the CFDA's prefectural branches. We utilize the features of prefectural CFDA websites as a basis to measure their transparency. The advantage of this approach is its objectivity and granularity, when compared with the prevailing survey methodology. The data can also be updated regularly.

The transparency of a prefectural CFDA branch is measured by the extent to which the agency discloses and solicits information related to food safety on its website. Worldwide, government websites are used to communicate with and receive information from the public. For example, the US Food and Drug Administration (FDA) provides information on food-borne illness alerts and product recalls on its website.^3^ This approach is useful especially for agencies that have dedicated functions and responsibilities.

**Fig. 1.**
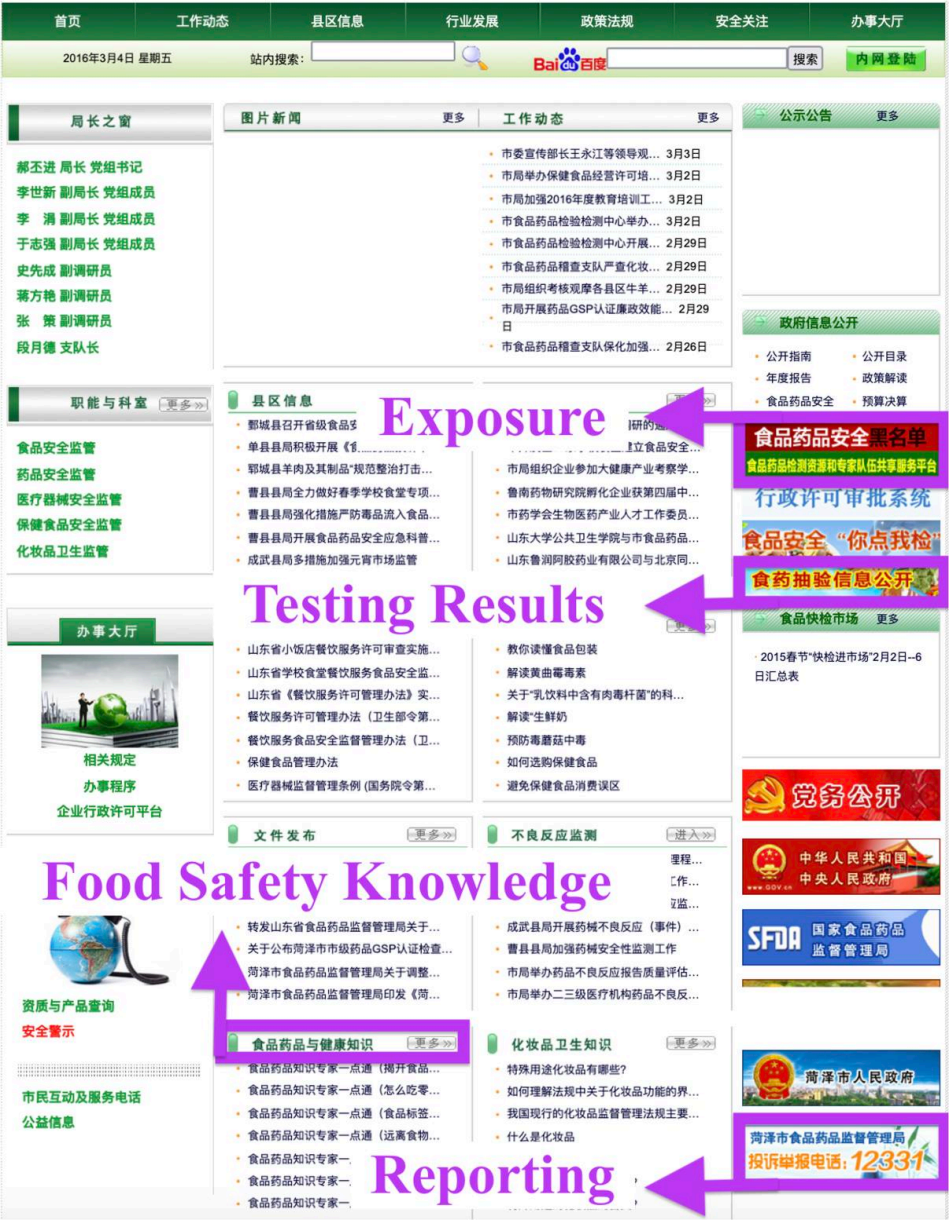
Screenshot of Heze prefecture's 2016 CFDA website (with annotation).

Our transparency measure includes four dimensions of the prefectural CFDA website: “Exposure,” “Reporting,” “Testing Results,” and “Food Safety Knowledge.” “Exposure” means blacklisting those companies that have violated food safety regulations. “Reporting” means providing a direct channel (e.g. email, phone, mailing address, or an online form) for the public to report food safety problems. “Testing Results” means publicizing the testing results of recent inspections on food manufacturers and products. “Food Safety Knowledge” refers to posting articles about food safety laws, regulations, and expert knowledge. We assign a score of 1 or 0 for each dimension based on whether that dimension is present on a prefectural CFDA website. A website can be scored 0–4 with 0 being the least transparent (i.e. none of the dimensions exist) and 4 being the most transparent (i.e. all four dimensions exist). In Fig. 1, we present a screenshot of Heze prefecture's 2016 CFDA website that has these four dimensions of regulatory transparency.

We use the Wayback Machine (https://archive.org/web/) to search for information about these four dimensions on the prefectural CFDA websites between 2008 and 2017. The Wayback Machine is the most comprehensive Internet archive which regularly stores the webpages of all websites on the World Wide Web. We develop an automated algorithm based on web scraping, text/image recognition, and string matching^4^ to calculate the transparency scores across a total of 2,226 archived webpages of the prefectural CFDA branches (see Supplementary Material S1). Based on manual verification on a random sample of 20% of the webpages, the accuracy of the algorithm is 88%.

**Fig. 2.**
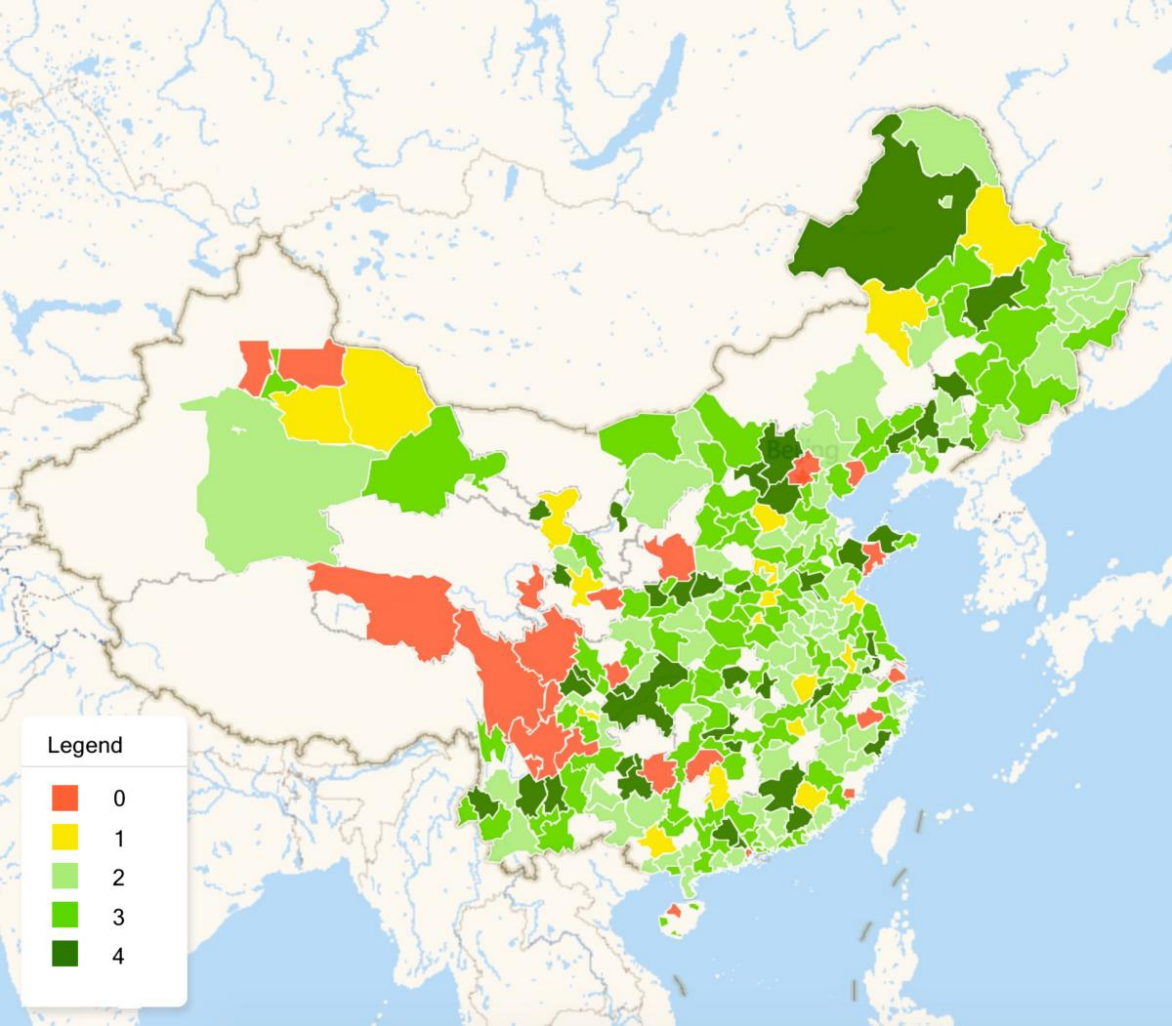
The prefectural CFDA transparency score map in 2013 (with imputed data). The figure presents the transparency scores of prefectural CFDA branches in 2013. The prefectures are color coded based on the transparency score of the prefectural CFDA: red = 0, yellow = 1, light green = 2, green = 3, dark green = 4. 272 prefectures are color coded. Prefectures without color are those for which the transparency score of the prefectural CFDA cannot be imputed in 2013. Prefectures that cannot be imputed are usually small in population and in remote regions. Lhasa of Tibet is an example.

In total, we obtain transparency scores for 298 out of 333 prefectural CFDA branches in China from 2008 to 2017 (see Missing Data section for a robustness test related to missing data). Our analysis is based on panel data organized at the prefecture-year level. Fig. 2 presents the transparency scores of prefectural CFDA branches in 2013 on a color-coded map. Red indicates low transparency and green indicates high transparency. The regional variations in the transparency of prefectural CFDA branches are evident in Fig. 2.

### Operationalizing CCP and administrative interventions

The Chinese state comprises of two arms. One runs through the party system and the other runs through the administrative system. The CFDA, now the State Administration for Market Regulation (SAMR), is headed by a party secretary and a director. (For the time frame of our data, the same person held both positions.) Each prefecture in China is headed by a party secretary and a mayor. There are some divisions of labor between the two heads of these government agencies, but usually the party secretary exercises more power and can use his or her veto power to overrule the administrative head of the government.

Food safety is a technocratic domain supervised by the CFDA until 2018 and by the SAMR thereafter. (Hereafter, we will refer to the food regulatory agency as the CFDA to simplify discussion.) The CFDA reports to the State Council (18), which is the apex of the administrative arm of the Chinese state. The CCP does not directly regulate the food sector (19). CCP's functions, as prominent as they are, are strategic, general, and non-specific to any industry domain. For this reason, most empirical studies on Chinese food safety regulation focuses on the administrative system (e.g. (3, 4, 6–13)), while overlooking the role of the CCP.

We operationalize the interventions by the CCP and the State Council by the actions these two organizations have taken respectively. The CCP intervention is operationalized as inspections of the provinces by the CCDI. The CCDI reports directly to the Politburo, the executive commission of the CCP, and its mission is to audit the conduct of government officials and CCP members to ensure their compliance with the directives of the CCP (20). The auditing covers a wide range of agencies, including state-owned enterprises. The mandate is broad, covering both conduct issues—e.g. corruption—and performance issues—e.g. failing to meet a target set by the central government. The general nature of the CCDI gives the agency a lot of power. The CCDI inspection teams can initiate investigations and impose punishments. They can show up unannounced, and they can be stationed in a government agency or in a locality for a period of time for observations and information collection.

During the time period of our data (2008–2017), the CCDI conducted one nationwide inspection, spanning from 2013 to 2014. This nationwide inspection was split into four rounds, with the list of provinces being inspected in each round summarized in Table S1 in Supplementary Material S2 (21). Given this inspection sequence, we define a dummy variable, Post_CCP_Intervene, at the prefecture-year level in the panel data. This variable is equal to 1 for a prefecture in an inspected province in the inspection year and thereafter; 0 otherwise.

The administrative intervention is based on actions taken by the State Council to establish FSCs in the Chinese provinces. In February 2010, the State Council established a central FSC, and in the subsequent period, the central FSC began to establish provincial FSCs. These FSCs are chaired by the vice provincial governors and are under the administrative arm of the Chinese state. We should note that the FSC is specifically empowered by the State Council to enforce food safety regulations. A review of the establishment notice of the FSC shows that it has the mandate to coordinate with law enforcement agencies, such as the Ministry of Public Security, the General Administration of Quality Supervision, Inspection and Quarantine, and the State Administration for Industry and Commerce. The central FSC reports directly to Li Keqiang, the premier of the State Council. The primary purpose of these FSCs is to better coordinate the actions of different local food regulatory and law agencies to improve food safety. Our a priori hypothesis is that this administrative intervention should have a significant impact on enhancing the transparency of the prefectural CFDA branches, especially when compared with a general-purpose intervention by the CCDI.

Similar as before, we construct a dummy variable, Post_Admin_Intervene, at the prefecture-year level in the panel data. This variable for a prefecture in a given year is equal to 1 in the year and thereafter when the FSC of the corresponding province has been established; 0 otherwise.

Out of 32 provinces, five set up their FSCs before 2008 as pilot programs prior to the establishment of the central FSC, and another one set up its FSC after 2017. Because the FSCs in these six provinces were established in the years outside of our time range, we perform robustness analysis by including or excluding data from these six provinces. The results remain consistent in both cases.

## Results

### Summary statistics

Table 1 shows the summary statistics on the number of observations, mean, standard deviation, and the minimum and maximum values for the prefectural CFDA transparency scores between 2008 and 2017. Average transparency scores across prefectures have increased over time.

**Table 1.** Summary statistics for the prefectural CFDA transparency scores by year.

Year	# Observations	Mean	Std.Dev.	Min	Max
2008	220	1.83	1.03	0	4
2009	217	1.94	1.01	0	4
2010	201	2.04	1.06	0	4
2011	231	2.21	1.08	0	4
2012	237	2.38	1.1	0	4
2013	253	2.45	1.1	0	4
2014	217	2.63	1.11	0	4
2015	226	2.91	1.09	0	4
2016	234	3.19	1.12	0	4
2017	194	3.56	0.8	0	4

### Impact of CCP actions on transparency

To examine the impact of CCP interventions on prefectural CFDA transparency, we estimate the following panel regression model as our baseline analysis:


(1) $$\mathrm{Transparenc}y_{i,t}=\;\beta_{0}+\beta_{1}\mathrm{Post}\_\mathrm{CCP}\_\mathrm{Interven}e_{i,t}+\beta_{2}\mathrm{GDPP}C_{i,t-1}+\beta_{3}\mathrm{MA}T_{i,t-1}+(\mathrm{prefecture},\;\mathrm{year}\;\mathrm{fixed}\;\mathrm{effects})+\varepsilon_{i,t}.$$

The subscripts *i* and *t* denote prefecture and year. The dependent variable, Transparency*_i,t_*, is the transparency score of the CFDA in prefecture *i* at year *t*. The key independent variable, Post_CCP_Intervene*_i_*_,*t*_, is a dummy variable that equals 1 for prefecture *i* for all years after the province containing prefecture *i* has been inspected by the CCDI, and it equals 0 for that prefecture for all years prior to the inspection. We exclude the prefecture-year observations during the year in which CCDI inspections were being conducted in the province where prefecture *i* is located. We control for GDP per capita (GDPPC*_i_*_,*t-1*_) and the marketization index (MAT*_i_*_,*t-1*_) at the prefecture-year level to account for regional and temporal variations in economic development. We also control for prefecture and year fixed effects. Since CCDI inspected all prefectures in China, we cannot use the difference-in-differences method. Instead, the inspection can be viewed as an exogenous shock to help us identify its impact on CFDA transparency.

Table 2 column (1) presents the baseline regression results. CCP interventions have a statistically significant, positive effect on the transparency of prefectural CFDA branches (*P* < 0.01). The increase in prefectural CFDA transparency (relative to the pre-CCDI-inspection period) is more substantial once the CCDI has inspected the corresponding province, when compared with those prefectures that have not been inspected by the CCDI. The magnitude of the increase is 1.74 on average, i.e. an increase of between 1 and 2 transparency scores, a substantial effect.

**Table 2.** The effect of CCP interventions on prefectural CFDA transparency.

Dependent variable	Transparency*_i_*_,*t*_	
	Model (1)	Model (2)
Post_CCP_Intervene*_i_*_,*t*_	1.74***	–
	(0.28)	
${\mathrm{Before}}_{i,t}^{-3}$	–	0.034
		(0.14)
${\mathrm{Before}}_{i,t}^{-2}$	–	0.26
		(0.24)
${\mathrm{Before}}_{i,t}^{-1}$	–	0.44
		(0.34)
${\mathrm{After}}_{i,t}^{1}$	–	1.72***
		(0.44)
${\mathrm{After}}_{i,t}^{2}$	–	1.66***
		(0.34)
${\mathrm{After}}_{i,t}^{3}$	–	1.71***
		(0.28)
${\mathrm{After}}_{i,t}^{4}$	–	1.80***
		(0.31)
GDPPC*_i_*_,*t*-1_	−0.15	−0.14
	(0.25)	(0.25)
MAT*_i_*_,*t*-1_	0.059	0.053
	(0.067)	(0.067)
Intercept	2.94	2.91
	(2.50)	(2.50)
Year dummy	Yes	Yes
Prefecture dummy	Yes	Yes
*N*	1,998	1,998
*R* ^2^	0.61	0.61

Robust standard errors adjusted for heteroskedasticity are clustered at the prefecture level. All models have controlled for prefecture and year fixed effects. *** indicate significance at the 1% level, respectively. “–” means the corresponding variable is not present in the model.

We also ask whether the effect of the CCP interventions on prefectural CFDA transparency is immediate or more gradual. To examine the potential dynamic changes of prefectural CFDA transparency pre- and post-CCP interventions, we estimate the following additional model:


(2) $$\mathrm{Transparenc}y_{i,t}=\;\beta_{0}+\beta_{1}{\mathrm{Before}}_{i,t}^{-3}+\beta_{2}{\mathrm{Before}}_{i,t}^{-2}+\beta_{3}{\mathrm{Before}}_{i,t}^{-1}+\beta_{4}{\mathrm{After}}_{i,t}^{1}+\beta_{5}{\mathrm{After}}_{i,t}^{2}+\beta_{6}{\mathrm{After}}_{i,t}^{3}+\beta_{7}{\mathrm{After}}_{i,t}^{4}+\mathrm{GDPP}C_{i,t-1}+\mathrm{MA}T_{i,t-1}+\left(\mathrm{prefecture},\;\mathrm{year}\;\mathrm{fixed}\;\mathrm{effects}\right)+\varepsilon_{i,t}.$$

The variable, ${\mathrm{Before}}_{i,t}^{-k}$ (with *k* = 1, 2, 3), is a dummy variable that equals 1 if the prefecture-year observation is from *k* years prior to CCDI's inspection of the corresponding province and 0 otherwise. Similarly, the variable, ${\mathrm{After}}_{i,t}^{k}$ (with *k* = 1, 2, 3, 4), is a dummy variable that equals 1 if the prefecture-year observation is from *k* years post CCDI of the corresponding province and 0 otherwise. All other variables are defined similarly as in model (1). The reference group in model (2) are observations from over 4 years prior to CCDI's inspection of a province.

**Fig. 3.**
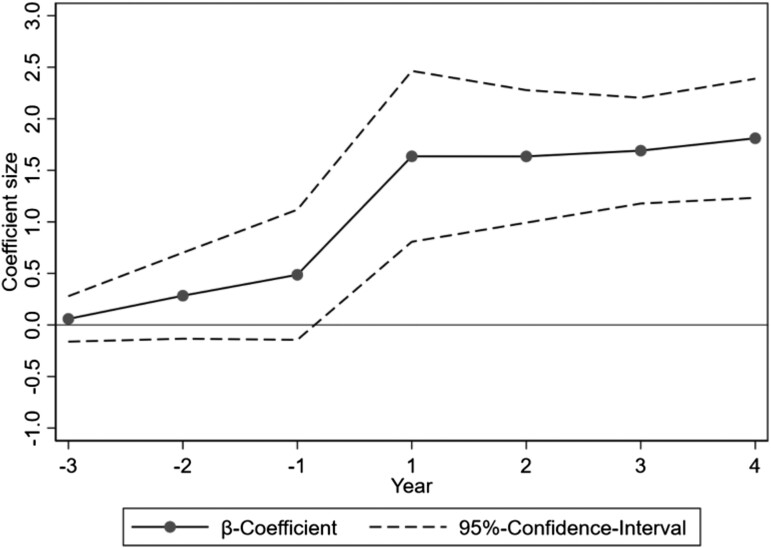
The temporal effect of CCP interventions on prefectural CFDA transparency. The figure shows the coefficient estimates and their 95% confidence intervals for the variables ${\mathrm{Before}}_{i,t}^{-k}$ (*k* = 1, 2, 3) and ${\mathrm{After}}_{i,t}^{k}$ (*k* = 1, 2, 3, 4) in model (2).

Table 2 column (2) presents the regression results of model (2). Fig. 3 shows the coefficient estimates and their 95% confidence intervals for the variables ${\mathrm{Before}}_{i,t}^{-k}$ (*k* = 1, 2, 3) and ${\mathrm{After}}_{i,t}^{k}$ (*k* = 1, 2, 3, 4). We observe that the statistically significant, positive effect of the CCDI inspection on prefectural CFDA transparency occurs immediately after the inspection, and this statistically significant transparency jump remains the same during the 4 years post inspection (with a moderate further increase in the fourth year post inspection).

### Impact of administrative actions on transparency

To examine the impact of administrative interventions on prefectural CFDA transparency, we estimate two panel regression models similar to models (1) and (2). The only difference is that the Post_CCP_Intervene*_i_*_,*t*_ variable in model (1) is replaced by the Post_Admin_Intervene*_i_*_,*t*_ variable. This new dummy variable equals 1 for prefecture *i* for all years after an FSC has been established in the province containing prefecture *i*, and it equals 0 for that prefecture for all years prior to the provincial FSC's establishment. In addition, the ${\mathrm{Before}}_{i,t}^{-k}$ (with *k* = 1, 2, 3) and ${\mathrm{After}}_{i,t}^{k}$ (*k* = 1, 2, 3, 4) variables in model (2) are defined based on the year in which the provincial FSC was established (instead of when CCDI's inspection of the province was conducted).

The regression results are presented in Table 3. We find no statistically significant results. Contrary to our a priori expectation, establishing a provincial FSC—the goal of which specifically focused on enhancing food-industry regulations—has *no* impact on changing the regulatory transparency of the prefectural CFDA branches.

**Table 3.** The effect of administrative interventions on prefectural CFDA transparency.

Dependent variable	Transparency*_i_*_,*t*_	
	Model (1)	Model (2)
Post_Admin_Intervene*_i_*_,*t*_	−0.12	–
	(0.22)	
${\mathrm{Before}}_{i,t}^{-3}$	–	−0.11
		(0.23)
${\mathrm{Before}}_{i,t}^{-2}$	–	−0.15
		(0.18)
${\mathrm{Before}}_{i,t}^{-1}$	–	0.092
		(0.16)
${\mathrm{After}}_{i,t}^{1}$	–	−0.065
		(0.15)
${\mathrm{After}}_{i,t}^{2}$	–	−0.076
		(0.16)
${\mathrm{After}}_{i,t}^{3}$	–	−0.097
		(0.15)
${\mathrm{After}}_{i,t}^{4}$	–	0.0077
		(0.12)
GDPPC*_i_*_,*t*-1_	−0.09	−0.068
	(0.28)	(0.28)
MAT*_i_*_,*t*-1_	0.013	0.012
	(0.067)	(0.069)
Intercept	2.68	2.54
	(2.76)	(2.83)
Year dummy	Yes	Yes
Prefecture dummy	Yes	Yes
*N*	1,809	1,809
*R* ^2^	0.59	0.59

Robust standard errors adjusted for heteroskedasticity are clustered at the prefecture level. All models have controlled for prefecture and year fixed effects. “–” means the corresponding variable is not present in the model.

### Ruling out confounding influences

#### Other central- and local-level administrative actions

One may ask whether there were food industry–related regulations or reforms led by the administrative system that overlapped with CCDI inspections in timing, which may have confounded the CCP interventions. The majority of FSCs were established between 2010 and 2016. The timing differs from that of the CCDI inspections which took place from 2013 to 2014. To further investigate, we performed a thorough review of all regulatory reforms related to the food industry led by the State Council in the last few decades. We focus on reforms at the national level to be comparable with CCDI inspections and FSC establishments, both of which are mandated by the central government. Given the top-down regulatory approach in China, it is very unlikely that a local administrative arm would initiate systemic reforms without central mandates. Table S2 in Supplementary Material S3 summarizes the history of China's food industry–related regulatory reforms. In addition to the establishment of FSCs, the only other national reform in recent years was the creation of the SAMR in 2018, a year after our data period. Thus, the possibility that the CCP interventions are confounded by concurrent food industry–related reforms led by the administrative system is minimal.

We also ruled out two other channels through which an administrative action could have confounded the effect of the CCDI inspections. One is that the CCP effect may have worked through the FSC channel rather than doing so independently. In model (1) of Table 4, we included both the CCP intervention and administrative intervention dummies, as well as an interaction term between the two. The only variable that remains significant is the CCP intervention dummy; neither the administrative intervention dummy nor the interaction term is significant. Hence, it is unlikely that the CCP effect occurs through the FSC channel. Second, one may argue that there has been a more general policy shift toward transparency in the Chinese government, which may confound the CCP effect we show. To test this possibility, we performed thorough archival research and find that, within the time period of our data, there was only one central mandate to increase transparency. This mandate was issued in 2011.^5^ We then include in our panel regression a new dummy variable, Post_2011, which is equal to 1 for all prefectures and all years after 2011, and 0 otherwise. In models (2) and (3) of Table 4, we observe that, after controlling for the timing of this central mandate, the effect of CCP interventions remains statistically significant as in our main models.

**Table 4.** The effect of CCP interventions on prefectural CFDA transparency: ruling out potential confounds from the administrative channel.

Dependent variable	Interaction effect	2011 effect		VAT/GDP ratio	
	Model (1)	Model (2)	Model (3)	Model (4)	Model (5)
Post_CCP_Intervene*_i_*_,*t*_	1.93***	1.13***		1.72***	
	(0.39)	(0.14)		(0.32)	
Post_Admin_Intervene*_i_*_,*t*_	−0.048				
	(0.12)				
Post_CCP_Intervene*_i_*_,*t*_ X	−0.16				
Post_Admin_Intervene*_i_*_,*t*_	(0.20)				
${\mathrm{Before}}_{i,t}^{-3}$			−0.022		−0.022
			(0.15)		(0.15)
${\mathrm{Before}}_{i,t}^{-2}$			0.21		0.21
			(0.26)		(0.26)
${\mathrm{Before}}_{i,t}^{-1}$			0.37		0.37
			(0.38)		(0.38)
${\mathrm{After}}_{i,t}^{1}$			1.44***		1.63***
			(0.42)		(0.50)
${\mathrm{After}}_{i,t}^{2}$			1.36***		1.55***
			(0.40)		(0.39)
${\mathrm{After}}_{i,t}^{3}$			1.48***		1.67***
			(0.43)		(0.32)
${\mathrm{After}}_{i,t}^{4}$			1.66***		1.86***
			(0.50)		(0.35)
GDPPC*_i_*_,*t*-1_	−0.023	−0.023	−0.039	−0.023	−0.039
	(0.29)	(0.29)	(0.29)	(0.29)	(0.29)
MAT*_i_*_,*t*-1_	−0.019	−0.019	−0.027	−0.019	−0.027
	(0.073)	(0.073)	(0.074)	(0.073)	(0.074)
VAT/GDP Ratio*_i_*_,*t*-1_	0.00039	0.00039	0.00039	0.00039	0.00039
	(0.00025)	(0.00025)	(0.00025)	(0.00025)	(0.00025)
Post_2011		0.58**	0.20		
		(0.25)	0.48		
Intercept	2.17	2.17	2.37	2.17	2.37
	(2.88)	(2.88)	(2.88)	(2.88)	(2.88)
Year dummy	Yes	Yes	Yes	Yes	Yes
Prefecture Dummy	Yes	Yes	Yes	Yes	Yes
*N*	1,764	1,764	1,764	1,764	1,764
*R* ^2^	0.57	0.57	0.57	0.57	0.57

Robust standard errors adjusted for heteroskedasticity are clustered at the prefecture level. All models have controlled for prefecture and year fixed effects. *** and ** indicate significance at the 1% and 5% level, respectively. “–” means the corresponding variable is not present in the model.

#### Administrative capacity

Is it possible that the CCP intervention works through the administrative capacity of the Chinese government? While this hypothesis does not invalidate our finding of the CCP effect, it can confound our findings. For example, the lack of the FSC effect can be due to the variances of administrative capacity across Chinese provinces, since the provincial FSCs are newly established and their effectiveness depends on prior investments in the administrative capacity of the corresponding provinces.

How to measure administrative capacity is nontrivial and worthy of research on its own. There does not exist a well-established measure in the literature, and data (un)availability is a serious constraint in constructing this measure. One, if imperfect, proxy is the collection of value-added tax (VAT). Previous literature suggests that VAT collection correlates with administrative capacity because VAT, unlike other types of taxes, is uniform across regions and most of the industries (at 17% of the value added) in China. Thus, any variations observed in the actual amount collected can be attributed to the local administrative capacity to assess tax liabilities and to collect tax revenues (22). In models (4) and (5) of Table 4, we control for the amount of VAT collection (as measured by the VAT to GDP ratio) at the prefecture-year level.^6^ We continue to observe a statistically significant effect of the CCP intervention.

#### Anti-corruption campaign

Is it possible that transparency response is a result of the anti-corruption campaign launched by Xi Jinping? If so, the CCP effect is not a general effect but one specific to the anti-corruption campaign. While CCDI is tasked with administering CCP's anti-corruption campaign, the mandate of the CCDI is broader, such as examining officials’ performance in advancing local economies and livelihoods in addition to auditing their conduct. To disentangle the impact of the broader CCDI inspections on prefectural CFDA transparency from that of the anti-corruption campaign, we perform the following analysis. Using data collected by Wang and Dickson (23) on the removal of local officials from their positions as a result of the anti-corruption campaign, we create two direct measures of the anti-corruption campaign: (i) the total number of officials removed in a prefecture, and (ii) the total weighted sum of officials removed in a prefecture, with the removal of a higher ranking official assigned a larger weight. The Wang and Dickson (23) data set includes 10 levels of Chinese bureaucrats where the highest level (state level) is coded as 1 and the lowest level (deputy township level) is coded as 10. We take the inverse of Wang and Dickson (23)'s coding as our weighted official removal measure. We then estimate three sets of panel regression models: (i) add either anti-corruption measures as a control variable in our main model (1) (see model (3)), (ii) add either measure and the interaction with the Post_CCP_Intervene variable as control variables in model (1) (see model (4)), and (iii) include either measure alone while removing the Post_CCP_Intervene variable from model (1) (see model (5)).


(3) $$\mathrm{Transparenc}y_{i,t}=\;\beta_{0}+\beta_{1}\mathrm{Post}\_\mathrm{CCP}\_\mathrm{Interven}e_{i,t}+\beta_{2}\mathrm{Official}\_\mathrm{Remova}l_{i,t}+\beta_{3}\mathrm{GDPP}C_{i,t-1}+\beta_{4}\mathrm{MA}T_{i,t-1}+(\mathrm{prefecture},\;\mathrm{year}\;\mathrm{fixed}\;\mathrm{effects})+\varepsilon_{i,t}.$$

(4) $$\mathrm{Transparenc}y_{i,t}=\;\beta_{0}+\beta_{1}\mathrm{Post}\_\mathrm{CCP}\_\mathrm{Interven}e_{i,t}+\beta_{2}\mathrm{Official}\_\mathrm{Remova}l_{i,t}+\beta_{3}\mathrm{Post}\_\mathrm{CCP}\_\mathrm{Interven}e_{i,t}\times \mathrm{Official}\_\mathrm{Remova}l_{i,t}+\beta_{4}\mathrm{GDPP}C_{i,t-1}+\beta_{5}\mathrm{MA}T_{i,t-1}+(\mathrm{prefecture},\;\mathrm{year}\;\mathrm{fixed}\;\mathrm{effects})+\varepsilon_{i,t}.$$

(5) $$\mathrm{Transparenc}y_{i,t}=\;\beta_{0}+\beta_{1}\mathrm{Official}\_\mathrm{Remova}l_{i,t}+\beta_{2}\mathrm{GDPP}C_{i,t-1}+\beta_{3}\mathrm{MA}T_{i,t-1}+(\mathrm{prefecture},\;\mathrm{year}\;\mathrm{fixed}\;\mathrm{effects})+\varepsilon_{i,t}.$$

Table 5 summarizes the regression results. We observe that the Post_CCP_Intervene variable remains significant and positive in models (3) and (4), and the anti-corruption campaign variable is never statistically significant in any of the models. These results show that the anti-corruption campaign by itself has no effect on prefectural CFDA transparency. Our measure of CCP interventions is unlikely to be confounded by CCP's anti-corruption campaign.

**Table 5.** The effect of CCP interventions on prefectural CFDA transparency: disentangling from anti-corruption campaign.

Dependent variable	Transparency*_i_*_,*t*_					
	# of removal			Weighted # of removal		
	(1)	(2)	(3)	(4)	(5)	(6)
Post_CCP_Intervene*_i_*_,*t*_	–	1.74***	1.74***	–	1.74***	1.74***
		(0.28)	(0.28)		(0.28)	(0.28)
Official_Removal*_i_*_,*t*_	0.00045	0.00045	0.00033	0.0019	0.0019	−0.021
	(0.0033)	(0.0033)	(0.013)	(0.027)	(0.027)	(0.12)
Post_CCP_Intervene*_i_*_,*t*_ ×	–	–	0.00013	–	–	0.024
Official_Removal*_i_*_,*t*_			(0.013)			(0.12)
GDPPC*_i_*_,*t*-1_	−0.15	−0.15	−0.15	−0.15	−0.15	−0.15
	(0.25)	(0.25)	(0.25)	(0.25)	(0.25)	(0.25)
MAT*_i_*_,*t*-1_	0.059	0.059	0.059	0.059	0.059	0.059
	(0.067)	(0.067)	(0.067)	(0.067)	(0.067)	(0.067)
Intercept	2.94	2.94	2.95	2.94	2.94	2.96
	(2.5)	(2.5)	(2.5)	(2.5)	(2.5)	(2.5)
*N*	1,998	1,998	1,998	1,998	1,998	1,998
*R* ^2^	0.61	0.61	0.61	0.61	0.61	0.61

Robust standard errors adjusted for heteroskedasticity are clustered at the prefecture level. All models have controlled for prefecture and year fixed effects. *** indicate significance at the 1% level, respectively. “–” means the corresponding variable is not present in the model.

### Robustness analyses

Here, we confirm the robustness of our findings through a number of additional analyses.

#### CFDA mergers

Beginning in 2015, several prefectures had experimented merging the prefectural CFDA branches with other local food regulatory agencies to improve food-industry regulations. To confirm the robustness of our findings, we exclude these prefectures from our data and re-estimate the main empirical models. We obtain the same results (Table 6, columns (1)–(4)).

**Table 6.** The effect of CCP and administrative interventions on prefectural CFDA transparency: robustness results.

Dependent variable	Transparency*_i_*_,*t*_									
	Excluding prefectures with CFDA merger				Imputed data				Placebo test	
	(1)	(2)	(3)	(4)	(5)	(6)	(7)	(8)	(9)	(10)
Post_CCP_Intervene*_i_*_,*t*_	1.78***	–	–	–	1.73***	–	–	–	–	–
	(0.28)				(0.25)					
Post_Admin_Intervene*_i_*_,*t*_	–	–	−0.11	–	–	–	−0.13	–	–	–
			(0.22)				(0.19)			
Post_Visit*_i_*_,*t*_	–	–	–	–	–	–	–	–	−0.011	–
									(0.10)	
${\mathrm{Before}}_{i,t}^{-3}$	–	0.039	–	−0.14	–	0.059	–	−0.051	–	−0.067
		(0.14)		(0.23)				(0.21)		(0.74)
${\mathrm{Before}}_{i,t}^{-2}$	–	0.26	–	−0.18	–	0.28	–	−0.12	–	−0.057
		(0.24)		(0.18)				(0.17)		(0.49)
${\mathrm{Before}}_{i,t}^{-1}$	–	0.45	–	0.078	–	0.49	–	0.099	–	0.13
		(0.35)		(0.16)				(0.15)		(0.99)
${\mathrm{After}}_{i,t}^{1}$	–	1.79***	–	−0.082	–	1.64***	–	−0.075	–	0.012
		(0.45)		(0.15)				(0.12)		(0.07)
${\mathrm{After}}_{i,t}^{2}$	–	1.73***	–	−0.092	–	1.64***	–	−0.067	–	−0.050
		(0.34)		(0.16)				(0.14)		(0.31)
${\mathrm{After}}_{i,t}^{3}$	–	1.76***	–	−0.11	–	1.69***	–	−0.076	–	−0.136
		(0.27)		(0.16)				(0.14)		(0.18)
${\mathrm{After}}_{i,t}^{4}$	–	1.80***	–	0.0042	–	1.81***	–	0.014	–	−0.13
		(0.31)		(0.13)				(0.11)		(0.18)
GDPPC*_i_*_,*t*-1_	−0.16	−0.15	−0.099	−0.074	−0.13	−0.14	−0.078	−0.055	−0.081	−0.075
	(0.11)	(0.25)	(0.28)	(0.28)	(0.12)	(0.09)	(0.27)	(0.27)	(0.081)	(0.08)
MAT*_i_*_,*t*-1_	0.063	0.056	0.018	0.016	0.055	0.048	0.020	0.019	0.037	0.0303
	(0.10)	(0.069)	(0.069)	(0.071)	(0.11)	(0.11)	(0.066)	(0.067)	(0.59)	(0.047)
Intercept	3.047	2.94	2.71	2.60	2.84	2.89	2.49	2.34	2.43	2.41
	(2.51)	(2.50)	(2.62)	(2.83)	(2.55)	(2.99)	(2.65)	(2.72)	(2.03)	(2.01)
*N*	1,977	1,977	1,787	1,787	2,210	2,210	1,973	1,973	2,279	2,279
*R* ^2^	0.61	0.61	0.60	0.60	0.61	0.61	0.60	0.60	0.61	0.61

Robust standard errors adjusted for heteroskedasticity are clustered at the prefecture level. All models have controlled for prefecture and year fixed effects. For column (10), the “Before” and “After” variables are defined based on the year of Premier Li's visit to a province. *** indicate significance at the 1% level, respectively. “–” means the corresponding variable is not present in the model.

#### Missing data

We do not have observations for every prefecture in every year. For example, a prefectural CFDA website does not exist, or the archived webpage cannot be opened. Prefectures with these website problems are usually small in population and in remote regions. Lhasa of Tibet is an example. These are treated as missing data in our main results. We perform a robustness test using imputed values for these missing observations. For example, if a prefectural CFDA website has a transparency score of 1 in 2009, but no observation in 2010 or 2011, and a transparency score of 4 in 2012, then we impute its transparency scores in 2010 and 2011 to be 2 and 3, respectively. Data missing in the first or the last year (i.e. 2008 and 2017) are still treated as no observation. Similar results are obtained using these imputed values (Table 6, columns (5)–(8)).

#### Placebo test against CCP interventions

We perform a placebo test against CCP interventions. We use provincial visits by Premier Li Keqiang, leader of the administrative system, as a placebo treatment. We re-estimate models (1) and (2) by replacing the Post_CCP_Intervene variable with a similarly defined Post_Visit variable for the visit by Premier Li. We observe no statistically significant effect of the Post_Visit variables (Table 6, columns (9) and (10)).

#### Falsification test against CCP interventions

We follow Karplus et al. (24) to perform a falsification test. In particular, we randomly generated five sets of hypothetical CCDI inspections and examine whether the prefectural CFDA transparency has changed after the timing of these hypothetical inspections. We re-estimate model (1) assuming the CCDI inspections occurred in the hypothetical years. We observe no statistically significant effect of the hypothetical inspection (Table 7).

**Table 7.** The effect of CCP interventions on prefectural CFDA transparency: falsification test.

Dependent variable	Transparency*_i_*_,*t*_				
	(1)	(2)	(3)	(4)	(5)
Post_CCP_Intervene*_i_*_,*t*_	0.13	0.17	−0.0061	−0.019	−0.18
	(0.85)	(0.22)	(0.05)	(0.12)	(0.15)
GDPPC*_i_*_,*t*-1_	−0.22	−0.17	−0.33	−0.3437	−0.18
	(0.71)	(0.58)	(1.05)	(0.32)	(0.60)
MAT*_i_*_,*t*-1_	−0.068	−0.0086	−0.0041	−0.0181	−0.015
	(0.40)	(0.05)	(0.02)	(0.10)	(0.09)
Intercept	4.37	3.56	5.13	5.31	3.73
	(4.28)	(4.09)	(4.46)	(4.57)	(2.09)
*N*	989	996	996	989	993
*R* ^2^	0.71	0.71	0.71	0.70	0.72

This table reports results from re-estimating model (1) assuming hypothetical CCDI inspection years with data from 2008 to 2012. The five columns correspond to five different sets of randomly generated hypothetical inspection years (all between 2009 and 2011). Robust standard errors adjusted for heteroskedasticity are clustered at the prefecture level. All models have controlled for prefecture and year fixed effects.

#### Food safety and the order of CCDI inspections

It is unlikely that the order of CCDI provincial inspections—our operationalization of CCP interventions—would be affected by the extent of food safety problems across provinces. We use food safety incident data to rule out this possibility. First, for each round of CCDI inspections, we compare the number of food safety problems per 10,000 people occurring in the prior year in the provinces inspected by CCDI versus those provinces not yet inspected, using *t* tests. Table 8, panel A shows that none of the comparisons are statistically significant. Second, we estimate the following logistic regression:


(6) $$\mathrm{Logit}(\mathrm{Inspectio}n_{\;j,t})=\beta_{0}+\beta_{1}\mathrm{Safet}y_{\;j,t-1}+\beta_{2}\mathrm{GDPP}C_{\;j,t-1}+\beta_{3}\mathrm{MA}T_{\;j,t-1}+(\mathrm{province},\;\mathrm{year}\;\mathrm{fixed}\;\mathrm{effects})+\varepsilon_{\;j,t}.$$

**Table 8.** Testing the relationship between food safety incidents and the order of CCDI inspections.

Panel A: Significance tests on number of food safety incidents per 10,000 people between provinces being inspected and provinces not yet inspected by the CCDI				
	Mean of Safety_*j*,*t*−1_			
Inspection round	Provinces being inspected	Provinces not yet inspected	Difference	*t*-value
1	0.21	0.27	−0.05	−0.43
2	0.22	0.28	−0.06	−0.40
3	0.26	0.29	−0.030	0.23

Panel B: Logistic regression results		
Dependent variable	Logit(Inspection_*j*,*t*_)	
$\mathrm{Safet}y_{\;j,t-1}$	−1.09	–
	(1.15)	
Three_year_avg	–	−4.71
		(3.09)
GDPPC*_j_*_,*t*-1_	0.82	−7.28
	(1.09)	(11.25)
MAT*_j_*_,*t*-1_	−0.10	−0.56
	(0.18)	(0.34)
Intercept	−8.26	82.46
	(10.67)	(120.39)
*N*	62	62
*R* ^2^	0.27	0.27

Standard errors are shown in the parentheses. “–” means the corresponding variable is not present in the model.

The subscript *j* denotes a province. The variable, Safety_*j*,*t*−1_, is the number of food safety incidents per 10,000 people occurring in province *j* 1 year prior to year *t*. The remaining variables are defined the same as before. We also estimate an alternative model that includes instead the average number of food safety incidents per 10,000 people occurring in province *j* across the 3 years prior to year *t*. Table 8, panel B summarizes the results and none of the coefficients associated with the food safety incident variables is statistically significant. Hence, there is no evidence that the order of CCDI inspections was affected by the extent of food safety problems across provinces.

Finally, we perform a robustness test to confirm the order of FSC establishments is not affected by the extent of food safety problems across provinces. Since most of the FSCs were established in 2011 (57%), we conduct a *t* test to test whether provinces that established FSCs in 2011 and provinces that established FSCs after 2011 differ significantly in terms of their food safety incidents in 2010, which is 1 year before 2011. The resulting *t* test has a *P*-value of 0.36, which suggests that the decision to establish FSC in 2011 or later is not affected by the extent of food safety problems in 2010.^7^

## Discussion and conclusions

CFDA—and now SAMR—regulates food companies as well as food markets. In the past 30 years, the Chinese food system has been a source of major food safety incidents and infectious animal disease outbreaks. The ensuing COVID-19 pandemic started from a seafood market in Wuhan. Systematic research on food safety regulations is more important than ever.

A comprehensive understanding of the Chinese food safety regulatory system needs to go beyond just focusing on the commonly known aspects of the Chinese system, such as the CFDA or SAMR and laws and regulations. We know that the role of the CCP is pertinent and important but to explicitly demonstrate it is challenging. In this paper, using an innovative approach, we show that the prefectural CFDAs’ transparency scores respond to actions by the CCP but not to actions by the State Council. This finding is noteworthy because the State Council's FSC mandate is directly targeted to enhancing food-industry regulations. Our results hold in a variety of specifications and a battery of robustness tests.

Our claim is not that the State Council—and its FSC— does not matter, but rather, in this important area of regulatory transparency, the actions taken by the CCP have a substantial impact as well. Due to a lack of data, we are not able to explain why the CCP is able to generate the kind of transparency impact shown in our analysis. One conjecture is related to the top-down nature of the Chinese system. Scholars have pointed out that “although food safety legislation is far reaching in China, the implementation of food safety laws is very difficult…[because] China has a broad administrative structure” (7). Transparency is a feature of the overall architecture of the Chinese system, unlike, for example, domain-specific issues such as testing for chemical compounds in food products. The CCP is probably more capable of implementing system-wide changes than the administrative arm of the government, and in recent years under Xi Jinping, the power of the CCP has increased (25). It is also possible that our findings are limited to food safety and may not be replicated in other industries. Food is a universal good, and it is possible that the CCP has a bigger operational role in an industry with this feature. That the CCP looms larger in system- and society-wide policy areas is a mere conjecture beyond the scope of this paper. More research is called for to assess this conjecture and the general implications of our findings.

We also make methodological contributions. Systematic research on regulatory systems is hampered by a lack of data, especially data about the opaque Chinese system. Existing empirical research on China relies heavily on survey data. In addition to the usual drawbacks with surveys (26–28), survey data are intermittent, domain generic, and often at a highly aggregate level. Furthermore, in the current political environment of China, survey research involving international collaboration is almost impossible. (The last survey by the World Bank related to the overall regulatory environment in China was conducted in 2005 and it only covered 120 cities. There were no questions about the food industry.) Our methodology has the potential to generate continuous and up-to-date regulatory data for China. (In a separate project, we are using the same methodology to create a regulatory measure for the Ministry of Agriculture, the agency in charge of regulating the upstream food industry.)

Finally, our findings suggest that the CCP has its own unique capacity to shape regulatory developments in food safety. This is a less understood aspect of the Chinese system and is less appreciated by outside analysts. A future research question is whether the regulatory impact of the CCP can translate into real effects on China's food safety. Our findings indicate that this is a promising research direction.^8^

## Supplementary Material

pgad028_Supplementary_Data

## Data Availability

The data availability statement is designed to be consistent with the PNAS Nexus guidelines. Specifically, data will be publicly released and shared. All data will be stored either in .csv format or .dta format. All codes that are used to produce the tables in this paper will be made available via OSF (https://osf.io/) in the format of .do file or .R file. At the time of publication, the following data sets will be made available via OSF (https://osf.io/): (i) Transparency scores of prefectural CFDAs between 2008 and 2017; (ii) Control variables for regressions in Tables 2–8, including prefecture-level GDP per capita, marketization index, and VAT to GDP ratio; and (iii) Wang, Yuhua; Dickson, Bruce, 2021, “Replication Data for: How Corruption Investigations Undermine Regime Support: Evidence from China,” https://doi.org/10.7910/DVN/DBA7TB, Harvard Dataverse.

## Footnotes

1 In fact, it appears that during the timeframe of our data set, both the party secretary and the director positions at a prefectural CFDA were always held by the same person. This is a more common practice during recent times. The transition from two different officials holding these two positions to the same person holding both positions took place before our time frame. Thus, statistically it is not relevant to our findings.

2 For example, Yongnian Zheng, a political scientist at the Chinese University of Hong Kong, once asserted CCP’s dominance in China by claiming CCP as the “organizational emperor” of China (2).

3 The US FDA has a webpage for food-borne illness alerts (https://www.fda.gov/food/recalls-outbreaks-emergencies/outbreaks-foodborne-illness) and one for product recalls (https://www.fda.gov/safety/recalls-market-withdrawals-safety-alerts).

4 We use Python web scraping packages including Selenium, Scrapy, and BeautifulSoup to access and collect data from the OCR API.

5 The document was published by the State Council under the name “Notice on the Priorities of Food Safety Tasks in 2011” (http://www.gov.cn/zwgk/2011-03/25/content_1831345.htm, accessed 2022 December 11).

6 The data source for the annual VAT data is China City Statistical Yearbooks.

7 Note that eight provinces established FSCs before 2011. However, we do not have data on provincial-level food safety incidents before 2010 and thus we are not able to run statistical tests on these eight provinces.

8 There is evidence that in areas important to the material welfare of the Chinese population, the CCP has taken actions that are beneficial. A recent study published in *PNAS* shows that the PM_2.5_ level declined significantly between 2013 and 2017 (13).
