# Supplementary material for: The Chinese Communist Party and regulatory transparency in China's food industry
Source: PNAS Nexus. 2023 Feb 3;2(3):pgad028. doi: 10.1093/pnasnexus/pgad028 (PMC10032355; doi:10.1093/pnasnexus/pgad028)
Supplement: pgad028_Supplementary_Data [file pgad028_supplementary_data.docx]

## Supplementary Material

### S1. Additional Details on the Transparency Scoring Method

We define four dimensions of transparency: “Exposure”, “Reporting”, “Testing Results” and “Food Safety Knowledge.” The keywords we used for each dimension are as follows: Exposure: “black list”, “red list”, “expose”, “administrative punishment” ["红黑榜", "黑名单", "曝光", "行政处罚"]; Report: “complaint”, “report”, “supervisory phone number”, “hotline” ["投诉", "举报", "监督电话", "监督热线", "服务热线"]; Test Results: “testing result”, “testing outcome”, “inspection result”, “inspection outcome” ["检验结果", "检测结果","抽检结果", "抽查结果", "抽样检验信息", "抽检信息","检测信息", "检验信息", "抽查公告", "抽查通告", "监督检查", "监督抽检", "监督抽查", "风险检测", "质量抽检", "质量抽查", "抽检公开", "抽查公开", "抽检公告", "抽检通告"]; Food Safety Knowledge: “food safety”, “food and drug safety” ["食品安全", "食药安全", "食品药品安全"]

To calculate the transparency score for the CFDA websites between 2008 and 2017, we devise an automated scoring program. The program can automatically read webpage links from Excel and then retrieve and score the content of the webpages. The automated program retrieves the webpage content in two ways: 1) obtaining the source content in the back end by system browser; 2) capturing the webpage and converting pictures into text by Baidu OCR function, which is an OCR API that can give the best results compared to peer OCR APIs. After the content on a webpage is retrieved, we first check the site status. We will skip a site if the webpage is down. For those webpages that function normally, we search for the keywords associated with the four dimensions of transparency and score the webpages accordingly. This process is repeated until all webpages are checked and scored. To test the robustness of the automated results, we manually score 20% of the CFDA webpages and compare with the automated results. The accuracy rate is 88%.

### S2. The 2013-2014 CCDI Inspections

The 2013- 2014 CCDI inspections were split into four rounds, summarized in *Table S1*.

| **Round #** | **Year** | **Provinces Inspected** |
| --- | --- | --- |
| 1 | 2013 | Chongqing, Guizhou, Hubei, Inner Mongolia, Jiangxi |
| 2 | 2013 | Anhui, Guangdong, Hunan, Jilin, Shanxi, Yunnan |
| 3 | 2014 | Beijing, Fujian, Gansu, Hainan, Henan, Liaoning, Ningxia, Shandong, Tianjin, Xinjiang |
| 4 | 2014 | Guangxi, Hebei, Heilongjiang, Jiangsu, Qinghai, Shaanxi, Shanghai, Sichuan, Xizang (Tibet), Zhejiang |

Table S1. The sequential order of CCDI inspections across provinces in 2013-2014

Source: China CCDI (2020).

### S3. Central-level Food Safety Regulatory Reforms in China

*Table S2* summarizes the history of food safety regulatory reforms led by the State Council in China.

| **Stage** | **Time Span** | **Administrative System** | **Main Regulatory Agency** | **Additional Regulatory Agencies** |
| --- | --- | --- | --- | --- |
| Hybrid transitional system | 1949*–*1952 | The coexistence of industrial self-regulation and governmental supervision | Ministry of Health | N/A |
|  | 1953-1978 |  | Related regulatory agencies | Ministry of Health |
|  | 1979*–*1993 |  | Ministry of Health | Regulatory agencies for food producers, regulatory agencies for agriculture, livestock farming and fishing, regulatory agencies for industry and commerce |
| Comprehensive external regulation | 1994*–*2002 | Dominated by external independent regulation | Ministry of Health | General Administration of Quality Supervision, Inspection and Quarantine, State Administration for Industry and Commerce, Ministry of Agriculture |
| Scientific supervisory system | 2003*–*2007 | Comprehensive coordination and segmented regulation | China Food and Drug Administration | General Administration of Quality Supervision, Inspection and Quarantine, Ministry of Health, Ministry of Agriculture, State Administration for Industry and Commerce |
|  | 2008*–*2010 |  | Ministry of Health | General Administration of Quality Supervision, Inspection and Quarantine, State Administration for Industry and Commerce, Ministry of Agriculture, China Food and Drug Administration |
|  | 2011*–*2012 |  | Food Safety Committee | General Administration of Quality Supervision, Inspection and Quarantine, State Administration for Industry and Commerce, Ministry of Agriculture, China Food and Drug Administration, Ministry of Health |
| Modernization of governance system | 2013*–*2018 | Trinary Regulatory System | Food Safety Committee | China Food and Drug Administration, State Administration for Industry and Commerce, General Administration of Quality Supervision, Inspection and Quarantine, Ministry of Agriculture |
|  | 2018*–*present |  | State Administration for Market Regulation | Ministry of Agriculture and Rural Affairs |

Table S2. The history of Chinese food industry related regulatory reforms (1949–2019)

Note. The content of this table is based on the information provided by Hu (2016) and Liu, Mutukumira and Chen (2019).
